# Roles of Short-Chain Fatty Acids in Inflammatory Bowel Disease

**DOI:** 10.3390/nu15204466

**Published:** 2023-10-21

**Authors:** Yoonhwa Shin, Sunhee Han, Juhui Kwon, Songhyun Ju, Tae Gyu Choi, Insug Kang, Sung Soo Kim

**Affiliations:** 1 Department of Biomedical Science, Graduate School, Kyung Hee University, Seoul 02447, Republic of Korea; jac03032@khu.ac.kr (Y.S.); sunheehan@khu.ac.kr (S.H.); kwonjh@khu.ac.kr (J.K.); thdgus8543@khu.ac.kr (S.J.); 2 Department of Biochemistry and Molecular Biology, School of Medicine, Kyung Hee University, Seoul 02447, Republic of Korea; chtag@khu.ac.kr; 3 Biomedical Science Institute, Kyung Hee University, Seoul 02447, Republic of Korea

**Keywords:** short-chain fatty acid, inflammatory bowel disease, microbiome, gut

## Abstract

The gut microbiome is a diverse bacterial community in the human gastrointestinal tract that plays important roles in a variety of biological processes. Short-chain fatty acids (SCFA) are produced through fermentation of dietary fiber. Certain microbes in the gut are responsible for producing SCFAs such as acetate, propionate and butyrate. An imbalance in gut microbiome diversity can lead to metabolic disorders and inflammation-related diseases. Changes in SCFA levels and associated microbiota were observed in IBD, suggesting an association between SCFAs and disease. The gut microbiota and SCFAs affect reactive oxygen species (ROS) associated with IBD. Gut microbes and SCFAs are closely related to IBD, and it is important to study them further.

## 1. Introduction

The human microbiome is a complex and diverse community of symbiotic microorganisms inhabiting the human body. It comprises trillions of bacteria, fungi, and viruses that coexist within the gastrointestinal tract, forming a mutually beneficial relationship with their host [1,2,3]. Despite its profound impact on human health and the underlying causes of diseases, comprehensive research on the gut microbiota has only gained momentum in the last two decades [4,5]. Recent revelations have unveiled that the influence of the gut microbiota extends beyond the gastrointestinal tract, encompassing the central nervous system, immune function, and even drug metabolism and absorption [6,7,8,9,10].

Short-chain fatty acid (SCFA), a key microbial metabolite produced by bacteria fermenting dietary fiber, is primarily used by colon cells as an energy source [11].

Current research shows that SCFAs play an important role in the intestines as they are produced by intestinal microorganisms, and SCFA levels are known to affect intestinal diseases such as IBD.

In this review, we will discuss how SCFAs are related to IBD and possible treatments using them.

## 2. Short-Chain Fatty Acid (SCFA)

SCFAs are fatty acids with fewer than six carbon atoms [8]. They can be created spontaneously in the liver via host metabolic pathways, but they are mostly produced by the gut bacteria when undigested fiber and resistant starch are fermented [12]. The names of the SCFAs and their formulas are presented in Table 1.

### 2.1. Metabolism and Peripheral Effects of SCFAs

The glycolytic route is the most prevalent way for bacteria to produce SCFAs. Certain bacteria, such as Bifidobacteria, may use the pentose phosphate route to create the same chemicals. Other factors that influence SCFA production include microbial species diversity, absolute microbial abundances in the host’s gut, and the time it takes food to pass through the gut [13,14,15].

Increased mobility through the colon may lower SCFA synthesis by reducing the amount of substrate available for microbial fermentation [16]. Furthermore, alterations in substrate availability caused by changes in intestinal transit time may alter microbial SCFA synthesis by influencing microbial composition and concentration [17,18].

SCFAs are a primary source of energy for colon cells and have a significant impact on intestinal homeostasis, energy metabolism, and immune response modulation. Acetate, propionate, and butyrate are the most frequent SCFAs in the stomach. Acetate is required for energy generation and lipid synthesis, whereas propionate is mainly involved in production of glucose in the liver and small intestine and butyrate is the preferred energy source for colonic lining cells [19,20]. Some studies have shown that elevated acetate levels may contribute to fat storage and are therefore linked to obesity. Studies have shown that increased acetate production activates the parasympathetic nervous system and increases ghrelin secretion and GSIS. This creates a positive feedback loop, resulting in hyperlipidemia, hypertriglyceridemia, ectopic lipid deposition in the liver and skeletal muscle, and liver and muscle insulin resistance [21].

Notably, butyrate also functions as a signaling molecule, inhibiting histone deacetylase activity and acting as a ligand for the GPR41 or GPR43 receptors [22,23]. This dual role of butyrate enhances the barrier function of intestinal epithelial cells and possesses anti-inflammatory properties. SCFA receptors include G protein-coupled receptors (GPRs). GPR43 is recognized by enteroendocrine L cells and the receptor is activated primarily by acetate and propionate. When expressed in adipose tissue, GPR43 plays a role in regulating lipid homeostasis and insulin, and when expressed in immune cells, it plays an anti-inflammatory role by increasing Treg cells and IL-10 [24,25]. GPR109A has also been identified as an SCFA receptor. These receptors exert different effects depending on their location. GPR109A is expressed by colonic epithelial cells, and when activated by (β-hydroxy-)butyrate, mediates anti-inflammatory properties such as IL-10 production. GPR41 is preferentially activated by propionate and butyrate instead of acetate. GPR41 induces PYY and GLP 1 secretion by L cells, which affects multiple tissues, including the cardiovascular system, pancreas, and brain [25,26,27].

### 2.2. SCFAs and Gut Metabolism

Short-chain fatty acids (SCFAs) play a vital role in gut metabolism, as they are produced through the fermentation of dietary fibers by the gut microbiota and subsequently serve as an important energy source for the cells lining the intestinal tract. The process of SCFA production and their interaction with gut metabolism is complex and involves various biochemical pathways [17].

Dietary fibers, which are complex carbohydrates that humans cannot digest, reach the colon relatively unchanged. In the colon, the gut microbiota, which is a diverse community of microorganisms, ferment these fibers [28]. This fermentation process breaks down the fibers into simpler compounds, including SCFAs. The primary SCFAs produced in the gut are acetate, propionate, and butyrate [29]. Bacteria in the colon convert various substrates derived from fiber fermentation into these SCFAs. Acetate is usually the most abundant SCFA, followed by propionate and butyrate. Once produced, SCFAs are absorbed by the cells lining the colon, called colonic epithelial cells [30]. These cells utilize SCFAs for energy through a process called oxidation. Butyrate, in particular, is a preferred energy source for colonic epithelial cells. Colonic epithelial cells metabolize SCFAs via several pathways, including beta-oxidation and the citric acid cycle (Krebs cycle). This metabolic process generates adenosine triphosphate (ATP), the cell’s primary energy currency. SCFAs contribute significantly to the energy needs of these cells, supporting their proper function and maintenance [31].

Butyrate is one of the most important SCFAs for gut health. It serves as the primary energy source for colonocytes (cells of the colon), promoting their growth and maintaining the integrity of the gut barrier. Butyrate also plays a role in gene expression regulation, apoptosis (programmed cell death), and the production of mucins, which are essential for the protective mucus layer in the intestines [32]. Propionate is primarily produced in the colon as a result of bacterial fermentation of complex carbohydrates and dietary fiber that are not digested in the small intestine [8]. Once produced, propionate is absorbed from the colon into the bloodstream. It enters the portal circulation, which carries it to the liver. In the liver, propionate undergoes further metabolic processing. It can be converted into glucose through a process called gluconeogenesis. This is an important mechanism for maintaining blood glucose levels, especially during fasting or low carbohydrate intake [33].

SCFAs have broader metabolic effects beyond the gut. For instance, propionate can be absorbed into the bloodstream and influence glucose metabolism and appetite regulation. Some SCFAs also interact with specific receptors on immune cells, regulating immune responses [31].

The interaction between SCFAs and gut metabolism is a part of the intricate crosstalk between the gut microbiota and the host. The composition of the gut microbiota and the availability of substrates influence the production and levels of SCFAs, which in turn affect gut health, metabolism, and even systemic effects in the body. 

SCFAs bolster intestinal barrier integrity through several mechanisms, including the induction of IL-18 secretion, antimicrobial peptide release, and mucin production by intestinal epithelial cells. Additionally, they upregulate the expression of tight-junction proteins, fortifying the physical barrier against pathogens. In response to inflammation, SCFAs promote neutrophil migration to sites of infection and enhance phagocytic activity, aiding in the clearance of pathogens. They also modulate T-cell function, primarily by inhibiting histone deacetylases (HDACs) and activating protein-coupled receptor (GPCR) pathways. This regulation extends to dendritic cells (DCs), where SCFAs influence T-cell differentiation. Furthermore, SCFAs directly impact T cells, contributing to their differentiation into various subsets, including Th1, Th17, and Tregs, within a complex cytokine milieu. Moreover, SCFAs temper the production of pro-inflammatory cytokines by intestinal macrophages and stimulate the production of intestinal IgA by B cells through HDAC inhibition. This immunoglobulin helps maintain immune tolerance in the gut. SCFAs inhibit carcinogenesis by promoting apoptosis (programmed cell death) and curbing the proliferation of tumor cells [29,31,32].

## 3. Gut Microbiome

The human microbiome is a diverse community of symbiotic bacteria that live in the human body. These germs infect the skin and other mucosal cavities, including the nasal cavity, oral cavity, and vagina. The gastrointestinal system (GI) has the highest density of these habitats [1,2]. Gut microbes are required for the fermentation of indigestible substrates such as dietary fiber. This enables the development of specialized bacteria that generate SCFAs. The microorganisms mostly create acetate, propionate, and butyrate [34].

In the onset and development of obesity, the gut microbiota appears to be crucial. The majority of overweight or obese people have dysbiosis, a condition marked by a loss of gut microbiota diversity [35]. Ley and colleagues, utilizing 16S rRNA gene sequencing, identified a decreased prevalence of the Bacteroidetes phylum and a significant increase in Firmicutes levels within an obese mouse model lacking leptin (ob/ob) [36]. Several months later, Turnbaugh, part of the same research team, corroborated these findings by comparing the Firmicutes-to-Bacteroidetes ratio in cecal bacterial DNA of obese mice from this model to that of lean, healthy mice, employing shotgun metagenomic sequencing. Additionally, the ob/ob mice displayed elevated levels of Archaea within their cecal microbial community relative to control mice [37]. These alterations in bacterial abundance prompted more extensive investigations into gut microbiota in other obesity models and in humans. Consequently, other studies related to obesity have revealed associations with increased levels of specific bacteria, such as Halomonas or Sphingomonas, as well as a reduction in Bifidobacteria [38].

**Figure 1:**
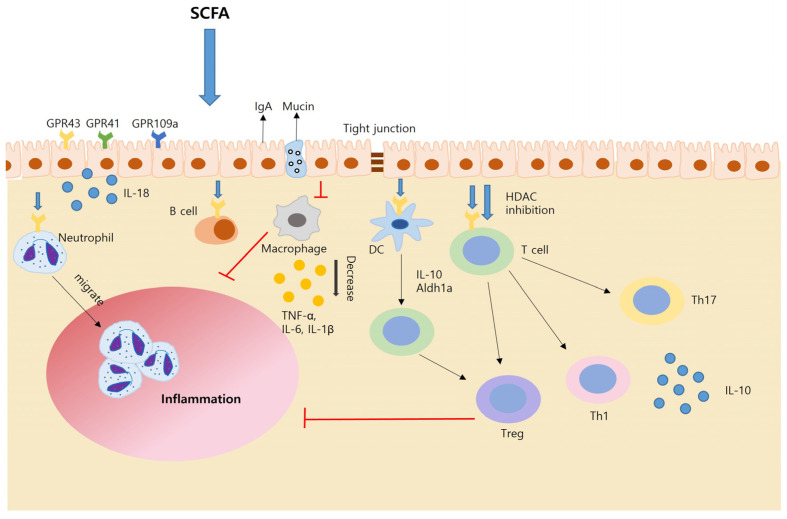
SCFA and overall flow on gut immunity.

While the gut microbiota composition is relatively diverse in healthy individuals, those with high adiposity, insulin resistance, and dyslipidemia, which are characteristic of obese patients, are linked to a lower bacterial gene count [4], indicating a comparatively less diverse gut microbiota. Obese patients have also exhibited a diminished proportion of Bacteroidetes and elevated Firmicutes levels (refer to Figure 1) [5,8,9]. Through a number of processes involving immune system dysregulation and inflammatory signaling pathways, this imbalance of gut microorganisms can cause obesity and other metabolic problems [39,40,41]. Additionally, it has been found that patients with inflammatory bowel disease, atopic eczema, and diabetes have less bacterial diversity than healthy control groups [42,43]. A “healthy gut” is often thought to be indicated by a diversity of bacterial species. Different types of gut microorganisms can be impacted by specific foods and diets, which may have further effects on your health [43].

Humans and other organisms have evolved in close association with a complex microbial community. These microorganisms, collectively known as the microflora, colonize nearly all exposed surfaces of the human body. One of the most diverse and abundant populations of microflora resides in the gastrointestinal tract, forming the intestinal microflora. The gut microbiota of bacteria is estimated to possess an astounding number of over 200 million genes, almost on par with the total count of human cells in the body. This intricate microbial community plays a pivotal role in regulating a wide range of biological processes by acting as a dynamic modulator and filter for various chemical signals originating from the environment. Consequently, the composition of the intestinal flora exerts a profound influence on human health and overall well-being [44].

A healthy gut microbiota helps to maintain BBB (blood–brain barrier) integrity by regulating tight-junction protein expression via short-chain fatty acids (SCFAs) [45,46]. SCFAs help to preserve intestinal barrier integrity by reducing microbial translocations that are linked to local intestinal and systemic inflammations and neuroinflammation [47,48]. However, microbiome dysbiosis, which is associated with an increase in potentially harmful bacteria, can alter the immune response by bacterial production of endotoxins, e.g., lipopolysaccharide (LPS), which can directly damage intestinal epithelial cells [49], impair inflammation, and intestinal barrier integrity. LPS interacts with immune cells in the bloodstream, upregulating systemic expression of inflammation [49] and proinflammatory cytokines such as TNF and interleukins, and at high quantities can be caused by direct breakdown of the BBB [50].

Within a day, dietary modifications can change the gut microbiota’s makeup, and even little adjustments to parameters like fiber content can have a positive impact on the microbiome [51]. The majority of nutrients in the Western diet, which is abundant in fat and digestible carbohydrates, are absorbed in the duodenum, which leaves the gut bacteria with insufficient substrates [52]. This makes the host more vulnerable to inflammatory disorders, including inflammatory bowel disease or colon cancer, as well as intestinal dysbiosis, compromised microbiota composition, and other inflammatory conditions.

### 3.1. SCFAs Producers in Gut

There are roughly 10^14^ of bacteria throughout the gastrointestinal tract, with denser colonies developing in the large intestine or colon. In addition to having a big impact on gut health, the colonic microbiome’s makeup and metabolism also have an impact on the host’s general health. While the majority of the bacteria are obligate anaerobes, the bacterial community of the gastrointestinal tract differs substantially from person to person [53].

**Figure 2:**
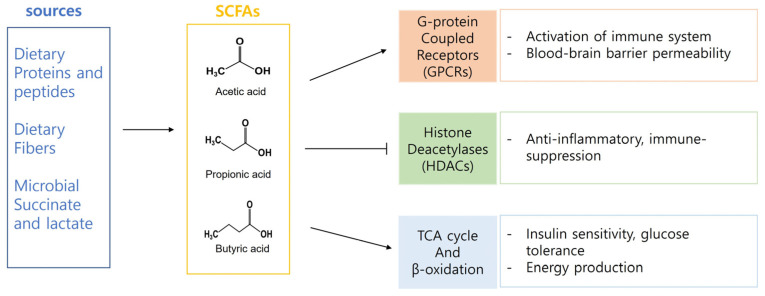
SCFAs are important tissue-specific energy and signaling molecules.

The gut microbiota, a complex and diverse microbial community residing in the gastrointestinal tract, plays an essential role in human health and disease [54,55]. One of the essential functions of gut microbiota is the production of short-chain fatty acids (SCFAs), which are organic acids with fewer than six carbon atoms. SCFAs, including acetate, propionate, and butyrate, are produced by the gut microbiota via the fermentation of dietary fibers and other indigestible carbohydrates (refer to Figure 2) [8,11].

The production of SCFAs is attributed to a variety of microorganisms present in the gut, including Firmicutes, Bacteroidetes, Actinobacteria, and Verrucomicrobia phyla [30]. Members of the Firmicutes phylum, particularly the Clostridia class, are considered the most efficient producers of butyrate, one of the most important SCFAs [56]. Examples of butyrate-producing bacteria within the Firmicutes phylum include *Faecalibacterium prausnitzii*, *Clostridium leptum*, *Eubacterium rectale*, and *Roseburia* spp. [57,58].

The Bacteroidetes phylum is also an important producer of SCFAs, particularly propionate [59]. This phylum can break down complex polysaccharides and generate a wide range of other metabolites [60]. Members of the Actinobacteria phylum, such as Bifidobacterium spp., can produce acetate, lactate, and other SCFAs in significant quantities [61,62]. *Akkermansia muciniphila*, a member of the *Verrucomicrobia* phylum, is known to produce both propionate and acetate and is considered very efficient in producing butyrate [63,64]. *A. muciniphila* is known to colonize the mucosal layer of the human intestine and trigger metabolic and immune responses in the host. *A. muciniphila* is particularly effective in increasing mucus thickness and increasing intestinal barrier function [65].

Moreover, different microorganisms express the genes encoding enzymes involved in butyrate synthesis, such as butyryl-CoA dehydrogenase, butyryl-CoA transferase, and/or butyrate kinase [66,67]. The expression of these genes could allow other potential producers to synthesize butyrate, including some members of the Proteobacteria, Spirochaetes, and Fusobacteria phyla, which are not commonly considered significant SCFA producers [30,68].

Taken together, the gut microbiota, composed of different phyla of microorganisms, plays a vital role in producing SCFAs, which have important implications for human health [34,43].

### 3.2. Effect of Diet on the Microbiome–SCFA Axis

#### 3.2.1. Ketogenic Diet

The ketogenic diet is a high-fat, moderate-protein, low-carbohydrate diet that has risen in popularity in recent years due to its possible health benefits, which include weight loss, better metabolic health, and neurological diseases. According to emerging research, the ketogenic diet may have an effect on the gut flora [69].

The ketogenic diet has been proven in studies to affect the composition of the gut microbiota. It has been demonstrated that the diet increases the abundance of certain bacterial species, such as *Akkermansia muciniphila*, while decreasing the abundance of others, such as Firmicutes [70]. Changes in the composition of the gut microbiome have been linked to improved metabolic health, lower inflammation, and improved intestinal barrier function [71,72,73].

The ketogenic diet may also have an impact on the formation of short-chain fatty acids (SCFAs), which are key metabolites produced by gut bacteria and have a variety of physiological effects, including preserving intestinal integrity, regulating immunological function, and moderating inflammation [74,75,76]. The ketogenic diet has been found in studies to enhance the production of certain SCFAs, such as beta-hydroxybutyrate (βHB), while decreasing the production of others, such as acetate and propionate. These alterations in SCFA synthesis could have consequences for gut and metabolic health [77,78].

Overall, current evidence suggests that the ketogenic diet may have an effect on the gut microbiome and its related health advantages, while additional study is required in this area. It is crucial to remember that the ketogenic diet may not be right for everyone and that its long-term impacts on gut health and general health are not yet fully understood.

#### 3.2.2. Mediterranean Diet

The Mediterranean diet is defined by high consumption of plant-based foods rich in fiber, including fruits, vegetables, legumes, and nuts. It also involves generous consumption of olive oil and seafood, while limiting the intake of red meat and sugary foods [79,80].

A study by De Filippis offered the initial solid proof of the intricate relationship among Mediterranean dietary patterns, gut microbiota, and microbial metabolites. Notably, they found that individuals adhering to the Mediterranean diet (MD) with significant consumption of fruits, vegetables, and legumes exhibited higher fecal SCFA levels. These SCFAs, in all likelihood, are the result of the activity of specific bacteria belonging to both the Firmicutes and Bacteroidetes phyla, capable of breaking down carbohydrates that cannot be digested by the host. In contrast, those with lower adherence to the MD showed elevated urinary TMAO levels [81].

Higher MD compliance was associated with higher fecal SCFA levels only in omnivores. This suggests that the MD plays a crucial role in providing the necessary substrates and promoting the growth of catabolic microbes, supporting SCFA production, even within a primarily omnivorous diet [34,81].

Moreover, the microbiota associated with vegetable-based diets showed positive correlations with SCFA levels. Notably, *Prevotella* within the Bacteroidetes phylum and *Lachnospira* within the Firmicutes phylum emerged as prime candidates for fermenting carbohydrates, ultimately leading to increased SCFA production [17,81,82,83].

## 4. SCFA and Inflammatory Bowel Disease (IBD)

The relationship between short-chain fatty acids (SCFAs) and inflammatory bowel disease (IBD) is intricate, involving a complex interplay among gut microbiota, immune responses, and the integrity of the gut epithelial barrier [30,84].

IBD is a chronic intestinal disease that is generally classified into one of two subtypes: Crohn’s disease and ulcerative colitis. Ulcerative colitis is limited to the colon, and superficial mucosal inflammation may extend proximally in an adjacent manner and cause ulcers, severe bleeding, toxic megacolon, and fulminant colitis. In contrast, Crohn’s disease can affect all parts of the digestive tract, often in a discontinuous manner, and is characterized by transmural inflammation, which can lead to complications such as fibrous strictures, fistulas, and abscesses. The pathophysiology of IBD involves complex genetic, environmental, epithelial, microbial, and immunological factors [85,86].

Gut microbiota play a pivotal role in maintaining both gut health and immune function. In individuals with a healthy gut, beneficial bacteria within the colon ferment dietary fibers to produce SCFAs like acetate, propionate, and butyrate. These SCFAs contribute significantly to microbial balance and play a crucial role in regulating immune responses [84].

Butyrate, in particular, has been shown to have anti-inflammatory properties. It can modulate immune cell function and reduce the production of pro-inflammatory cytokines. SCFAs achieve this by interacting with specific receptors on immune cells, such as G protein-coupled receptors (GPCRs), to regulate immune responses in the gut [87].

In IBD, which includes conditions like Crohn’s disease and ulcerative colitis, there is chronic inflammation of the gastrointestinal tract. This inflammation can compromise the integrity of the gut epithelial barrier, allowing harmful substances to penetrate the intestinal lining and trigger immune responses. SCFAs contribute to the maintenance of this barrier by promoting the production of mucus and enhancing the tight junctions between epithelial cells [88]. IBD is characterized by an aberrant immune response where the immune system mistakenly attacks the gut lining, leading to chronic inflammation. SCFAs help regulate this immune response by influencing the differentiation and function of immune cells, such as regulatory T cells (Tregs), which help suppress excessive immune reactions [89]. Moreover, SCFAs participate in tissue repair processes within the gut. They promote the proliferation and differentiation of epithelial cells, aiding in the healing of damaged tissues caused by inflammation in IBD [30].

Previously, it has been indicated that individuals with IBD might have altered levels of SCFAs in their gut due to disruptions in gut microbiota composition and reduced fermentation of dietary fibers. This disruption can contribute to the dysregulation of the immune response and compromised gut barrier function seen in IBD [90]. It is important to note that while there is evidence supporting the potential beneficial effects of SCFAs in mitigating inflammation and maintaining gut health, the relationship between SCFAs and IBD is complex and not fully understood. The field of research into gut microbiota, SCFAs, and their role in various gastrointestinal conditions, including IBD is ongoing, and further studies are needed to better elucidate the mechanisms and potential therapeutic applications [17,28,29,30,31,32,87,90].

## 5. Treatment or Prospect

The idea that probiotic, prebiotic, and synbiotic supplementation modifies IBD symptoms and improves many of the disease-related biomarkers is supported by recent evidence.

### 5.1. Probiotics

Inflammatory bowel disease (IBD), which includes conditions like Crohn’s disease and ulcerative colitis, involves chronic inflammation of the gastrointestinal tract. Probiotics are live microorganisms that—when administered in adequate amounts—can provide health benefits to the host, particularly by modulating the gut microbiota and immune responses. The link between IBD and probiotics is a subject of ongoing research, and while there is potential for probiotics to offer benefits, the relationship is complex and context-dependent [91,92].

Individuals with IBD often exhibit an imbalance in their gut microbiota composition, known as dysbiosis. Dysbiosis can contribute to the inflammation characteristic of IBD. Probiotics are thought to help restore microbial balance by introducing beneficial bacteria to the gut, which could potentially counteract the overgrowth of harmful bacteria associated with IBD [93,94].

Probiotics have been shown to interact with the immune system, both locally in the gut and systemically. IBD involves an aberrant immune response where the immune system attacks the gut lining. Probiotics might help regulate this response by promoting the development of regulatory T cells (Tregs) and other immune cells that can dampen excessive inflammation [95,96,97].

Certain strains of probiotics have demonstrated anti-inflammatory properties. They can produce bioactive molecules, such as short-chain fatty acids (SCFAs), that have immune-modulating and anti-inflammatory effects. These molecules can potentially help mitigate the inflammation observed in IBD [92,95,96].

Probiotics might contribute to maintaining the integrity of the gut epithelial barrier. In IBD, this barrier can become compromised, allowing harmful substances to enter the intestinal tissue and trigger inflammation. Probiotics can strengthen the barrier by promoting the production of mucus, enhancing tight junctions between cells, and fostering a healthy gut lining [98,99].

Probiotic bacteria can produce various bioactive compounds, including vitamins, enzymes, and metabolites, that have beneficial effects on gut health. Some of these compounds might influence factors relevant to IBD, such as inflammation and tissue repair [100,101].

Clinical studies examining the effects of probiotics on IBD have yielded mixed results. While some studies suggest potential benefits, others show limited efficacy or even adverse effects. The effectiveness of probiotics likely depends on factors such as the specific strains used, the disease subtype, individual variations, and the timing of administration [102,103].

IBD is a heterogeneous disease, meaning it can manifest differently in different individuals. Similarly, the response to probiotics can vary. A personalized approach considering an individual’s microbiota composition, the strain of probiotics used, and the stage of the disease might be more effective in harnessing the potential benefits of probiotics for IBD management [104].

It is essential to note that while probiotics hold promise as a potential adjunct therapy for IBD, they should not replace standard medical treatments. Consulting a health-care professional before incorporating probiotics into an IBD management plan is crucial, as their use needs to be tailored to the individual’s specific condition and needs.

#### Recent Clinical Trials on Probiotics in IBD

Normalization of colon microbiome composition could potentially offer substantial advantages to individuals with IBD. Multiple probiotic strains have been investigated and could provide noteworthy benefits to IBD patients. The findings from these examinations are outlined in Table 2.

### 5.2. Prebiotics

IBD is often associated with an imbalance in the gut microbiota composition, known as dysbiosis. Dysbiosis can contribute to the inflammation characteristic of IBD. Prebiotics, being non-digestible fibers, reach the colon intact and serve as a nutrient source for beneficial gut bacteria. By promoting the growth of these bacteria, prebiotics could potentially help restore a more balanced gut microbiota composition [115].

Prebiotics are fermented by gut bacteria in the colon, leading to the production of short-chain fatty acids (SCFAs) as metabolic byproducts. SCFAs, particularly butyrate, have anti-inflammatory properties and play a role in maintaining the health of the gut lining. In IBD, where inflammation damages the gut barrier, the increased production of SCFAs due to prebiotic fermentation can support gut healing and reduce inflammation [116].

The gut microbiota has a significant impact on the immune system, and dysbiosis in IBD can contribute to immune dysfunction. Prebiotic-induced changes in the gut microbiota can influence the immune response by promoting the growth of beneficial bacteria that contribute to immune regulation and suppression of excessive inflammation.

IBD is associated with a compromised gut epithelial barrier, allowing harmful substances to penetrate the gut lining and trigger inflammation. Prebiotics can contribute to maintaining the integrity of the mucosal barrier by promoting the production of mucus and enhancing the function of tight junctions between gut epithelial cells [95,117].

Prebiotic fermentation leads to the production of various metabolites beyond SCFAs, including gases and other bioactive compounds. These metabolites can influence gut signaling pathways, immune responses, and other factors relevant to IBD pathogenesis [118].

Clinical studies investigating the effects of prebiotics on IBD are still in progress, and the results have been somewhat mixed. Some studies suggest that prebiotics might improve certain clinical parameters, such as reducing inflammation markers and improving gut symptoms. However, as with any dietary intervention, individual responses can vary, and more research is needed to establish consistent benefits [119,120].

IBD is a heterogeneous disease, and responses to dietary interventions like prebiotics can vary widely among individuals [121]. A personalized approach that considers an individual’s specific microbiota composition, disease subtype, and dietary habits is crucial to optimizing the potential benefits of prebiotics for IBD management.

It is important to note that while prebiotics hold promise as a potential dietary strategy for IBD management, they should be used as part of a comprehensive treatment plan that includes medical guidance and other appropriate therapies. Consulting a health-care professional before making dietary changes is essential, especially for individuals with IBD.

### 5.3. Possibilities of SCFAs as Therapeutics

Short-chain fatty acids (SCFAs) have emerged as a promising treatment strategy for inflammatory bowel disease (IBD), encompassing conditions like Crohn’s disease and ulcerative colitis [95].

In a previous study, Sodium propionate inhibited the downregulation of tight junction proteins such as ZO-1, occludin, and E-cadherin and improved DSS-induced intestinal barrier dysfunction. Additionally, sodium propionate reduced the expression of pro-inflammatory factors TNFα, IL-1β, and IL-6 mRNA in colonic tissue. And sodium propionate suppressed oxidative stress in the colon by reducing MPO activity and enhancing SOD and CAT activities in serum and colon [122].

Butyrate, propionate, and acetate have been shown to mitigate ethanol-induced intestinal epithelial tight junction barrier dysfunction in Caco-2 monolayers. Additionally, SCFAs partially prevented ethanol-induced cellular oxidative and metabolic stress as well as displacement of tight-junction proteins and stress fiber formation. We found that these beneficial effects of SCFAs were mediated by activation of the AMPK pathway [123].

Moreover, administration of P. pentosaceus LI05 increased the abundance of specific genera, such as *Akkermansia* and *Faecalibacterium*, and helped regulate gut microbiota, reduce host inflammation, and increase SCFA production [124]. SCFAs may reduce the risk of chronic colitis developing into colon cancer and play an antitumor role by promoting apoptosis of cancer cells. Studies have shown that the number of butyrate-producing bacteria in colon cancer patients is significantly reduced and the expression of receptors GPR43 and GPR109A is also significantly reduced, indicating that SCFAs have a protective effect on colitis and colon cancer [125].

The therapeutic potential of SCFAs for IBD is grounded in their capacity to modulate various aspects of gut health, immune responses, and inflammation. Butyrate in particular has demonstrated anti-inflammatory properties both in vitro and in animal studies [23,126]. They can inhibit the production of pro-inflammatory cytokines and chemokines, molecules that contribute to inflammation. By reducing these inflammatory mediators, SCFAs may help alleviate the chronic inflammation seen in IBD [89]. Dysregulated immune responses play a pivotal role in IBD pathogenesis. SCFAs can influence immune cell differentiation and function. They promote the development of regulatory T cells (Tregs) that help control immune reactions and suppress excessive inflammation. SCFAs can also impact other immune cells, such as dendritic cells and macrophages, potentially promoting a more balanced immune response [127].

The gut epithelial barrier, which separates the gut contents from the underlying tissue, is compromised in IBD. SCFAs play a role in maintaining the integrity of this barrier by promoting the production of mucus and enhancing tight junctions between gut epithelial cells. This can help prevent the entry of harmful substances into the intestinal tissue [128]. SCFAs, particularly butyrate, can promote the proliferation and differentiation of colonic epithelial cells, contributing to the repair of damaged tissue [30]. This is crucial in IBD, where the gut lining is often inflamed and injured. SCFAs are produced by the gut microbiota through the fermentation of dietary fibers [34]. Imbalances in the gut microbiota are associated with IBD. By promoting the growth of beneficial bacteria that produce SCFAs, their administration could help restore microbial balance, which in turn may contribute to reduced inflammation [129]. SCFAs can be administered orally or rectally. Rectal administration, such as through enemas, allows SCFAs to have direct contact with the inflamed intestinal mucosa, potentially providing more targeted effects [130]. IBD is a complex and heterogeneous disease, and responses to treatments can vary widely among individuals. An individual’s specific disease subtype, severity, microbiota composition, and other factors can influence the response to SCFA treatment [131]. Tailoring treatment to the individual’s needs is essential for optimizing outcomes.

While there is preclinical evidence and some clinical data suggesting the potential of SCFAs for IBD treatment, larger randomized controlled trials are needed to establish their efficacy and safety. Researchers are actively investigating different SCFA formulations, dosages, and administration routes to determine the most effective approach.

In summary, the potential of SCFAs as a treatment for IBD stems from their ability to modulate inflammation, immune responses, and gut barrier function. While findings are promising, more research is necessary to fully understand the mechanisms, optimal dosing, and long-term effects of SCFA-based therapies for IBD. It is important for individuals with IBD to work closely with health-care professionals when considering SCFA-based treatments as part of their management plan.

## 6. Conclusions

In this review, a substantial body of evidence is presented, highlighting the significant role of the gut microbiota in mitigating the progression of IBD. The intricate connection between the gut microbiota and IBD is well established. Numerous studies have showcased the therapeutic potential of probiotics or prebiotics administration in the management of IBD. Hence, the identification of disease-specific alterations in the gut microbiome is of utmost importance to devise more effective treatment strategies. Consequently, it is imperative to conduct further clinical trials in order to advance our understanding in this field.

## Figures and Tables

**Table 1:** List of short-chain fatty acids.

Number of Carbon Atoms	Common Name	Systematic Name	Molecular Formula	Structural Formula	Mass (g/mol)	Diagram
C1	Formic acid	Methanoic_6_ acid	CH_2_O_2_	HCOOH	46.03	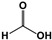
C2	Acetic acid	Ethanoic acid	C_2_H_4_O_2_	CH_3_COOH	60.05	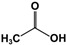
C3	Propionic acid	Propanoic acid	C_3_H_6_O_2_	CH_3_CH_2_COOH	74.08	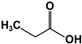
C4	Butyric acid	Butanoic acid	C_4_H_8_O_2_	CH_3_(CH_2_)_2_COOH	88.11	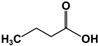
C4	Isobutyric acid	2-Methyl propanoic acid	C_4_H_8_O_2_	(CH_3_)_2_CHCOOH	88.11	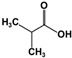
C4	Valeric acid	Pentanoic acid	C_5_H_10_O_2_	CH_3_(CH_2_)_3_COOH	102.13	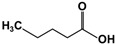
C5	Isovaleric acid	3-Methylbutanoic acid	C_5_H_10_O_2_	(CH_3_)_2_CHCH_2_COOH	102.13	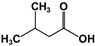
C5	2-Methylbutyric acid	2-Methylbutyric acid	C_5_H_10_O_2_	CH_3_CH_2_CH(CH_3_)COOH	102.13	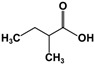

**Table 2:** List of recent clinical trials on probiotics in IBD.

Probiotic Used	Study	Number of Patients	Outcome
*Escherichia coli* Nissle 1917	Kruis W. et al., 1997 [105]	120	The capacity to maintain remission and stave off relapse is akin to mesalazine.
	Kruis W. et al., 2004 [106]	327	The capacity to maintain remission with a similar level of safety is akin to mesalazine.
	Matthes H. et al., 2010 [107]	90	The chance of achieving dose-dependent effectiveness in inducing remission with the rectal probiotic, as opposed to a placebo.
Bifidobacterium breve, Bifidobacterium bifidum, Lactobacillus acidophilus YIT 0168 (Bifidobacteria-Fermented Milk- BFM)	Ishikawa et al., 2003 [108]	21	Increased efficacy of the probiotic combination as an adjunct treatment in preserving remission and averting relapse when contrasted with conventional therapy in isolation.
	Kato K. et al., 2004 [109]	20	Increased effectiveness of the probiotic as supplementary treatment in sustaining remission when compared to conventional therapy alone.
	Matsuoka et al., 2018 [110]	195	No significant differences between groups; study discontinued
Lactobacillus casei, Lactobacillus plantarum, Lactobacillus acidophilus and Lactobacillus delbrueckii subsp. Bulgaricus, Bifidobacterium longum, Bifidobacterium breve and Bifidobacterium infantis, Streptococcus salivarius subsp. Thermophils (VSL#3)	Tursi A. et al., 2010 [111]	144	The probiotic mixture, when used as an additional treatment alongside conventional therapy, demonstrated superior effectiveness in patients with recurring conditions compared to a placebo.
	Sood A. et al., 2009 [112]	147	Superior efficacy in both inducing and sustaining remission in comparison to a placebo.
	Fedorak et al., 2015 [113]	120	After 90 days, there were no notable distinctions between the groups; however, in a one-year follow-up, a lower occurrence of severe endoscopic recurrence was observed in the VSL#3 group post-resection (*p* = 0.09). Additionally, the probiotic group showed a reduction in inflammatory cytokine levels after 90 days (*p* < 0.05).
Bifidobacterium longum 536	Tamaki et al., 2016 [114]	56	The study group showed a substantial enhancement in UCDAI (*p* < 0.01) and MAYO score, while the control group exhibited no improvements.
